# The Medical Certificate of the Cause of Death

**Published:** 1888-09

**Authors:** Augustin Prichard

**Affiliations:** Consulting Surgeon to the Bristol Royal Infirmary


THE BRISTOL

Medico-Chirurgical Journal.

SEPTEMBER, 1888.

### THE MEDICAL CERTIFICATE OF THE CAUSE OF DEATH.

BY

AUGUSTIN PRICHARD, F.R.C.S.,

*Consulting Surgeon to the Bristol Royal Infirmary.*

HAVING kept nearly all the "counterfoils" of the certificates of the cause of death since the beginning of my practice, many years ago, I thought it would be worth while to classify them and record the results, and thus give an idea respecting the nature and comparative numbers of the fatal cases met with in an average general practice, as well as some points of collateral interest which will have presented themselves in the course of my calculations; but I am afraid that my statement will be chiefly numerical, and, that although of some interest, I can hardly hope to extract much from it that will prove to be of scientific or practical value.

It must be borne in mind that this series contains only my own patients, and none of the many whom I have been called to see in consultation with other medical men, from whom the necessary certificates were obtained; and thus it is that cases of accident or hernia or urinary extravasation or other surgical fatalities are almost entirely absent: and although my practice has largely dealt with surgical cases, it will be noticed that only a very small minority appears here; and at the same time it must be remembered that they are more curable than those usually described as medical.*

The entire number is 426—that is, about ten for each year, supposing the years to have been equally busy, which, of course, was very far from being the case, and I have arranged them in ten tables, the first of which gives the proportionate numbers as to sex and age, and the others record the diseases, for the most part in the order of the "Nomenclature of Disease." I have added a few remarks after each, and have avoided as much as possible any reference to symptoms or treatment, as being foreign to a paper intended to be entirely pathological.

TABLE No. I.

| Ages. | No. | Males. | Fem. | Percentage. Males. | Percentage. Fem. |
|---|---|---|---|---|---|
| Under 3 months ... ... | 10 | 5 | 5 | 50 | 50 |
| 3 to 12 months ... ... | 7 | 4 | 3 | 57 | 43 |
| 1 to 2 years ... ... | 10 | 5 | 5 | 50 | 50 |
| 2 to 5 ,, ... ... | 15 | 8 | 7 | 53 | 47 |
| 5 to 10 ,, ... ... | 11 | 7 | 4 | 64 | 36 |
| 10 to 20 ,, ... ... | 24 | 11 | 13 | 46 | 54 |
| 20 to 30 ,, ... ... | 15 | 6 | 9 | 40 | 60 |
| 30 to 40 ,, ... ... | 18 | 6 | 12 | 33 | 67 |
| 40 to 50 ,, ... ... | 33 | 20 | 13 | 61 | 39 |
| 50 to 60 ,, ... ... | 48 | 18 | 30 | 38 | 62 |
| 60 to 70 ,, ... ... | 78 | 30 | 48 | 38 | 62 |
| 70 to 80 ,, ... ... | 90 | 34 | 56 | 38 | 62 |
| 80 to 90 ,, ... .. | 56 | 13 | 43 | 24 | 76 |
| 90 to 100 ,, ... ... | 11 | 3 | 8 | 27 | 73 |
| | 426 | 170 | 256 | 39.5 | 60.5 |

* These calculations do not include any deaths during the last three years.

From this table it appears that out of the whole number 170 were males and 256 were females, or as nearly as possible two to three, which appears a large difference: but we must bear in mind the fact that where I live, and have been living for three quarters of the time under consideration, the disparity of the sexes is almost, if not quite, the largest in England; for, according to the census of 1871, in all England the numbers were 48.5 males to 51.5 females, in the Clifton Union they were 44 to 55, in the St. Paul's (Clifton) district the disparity was even greater, and in my certificates it was 40 to 60.

In putting together the materials for this table an interesting point appeared, which however is not more than might be expected, viz., that as I have grown old, so have my patients, and whereas the average age at which the first fifty died was 28, the average age of the last fifty was 64, a much greater difference than one would have anticipated: and of those who died under 50 years of age, the number of males and females was nearly equal, *i.e.* 72 males to 71 females; while above 50 years of age the number of the women was nearly double that of the men, viz., 185 to 98. The average age of all was 54, and as many as 157 out of 426 died above the age of 70.

TABLE No. II.

| | |
|---|---|
| General Diseases ... ... ... ... ... ... ... ... | 54 |
| Diseases of the Nervous System ... ... ... | 72 |
| ,, ,, Circulating System ... ... ... | 32 |
| ,, ,, Respiratory ,, ... ... ... | 108 |
| ,, ,, Digestive ,, ... ... ... | 57 |
| ,, ,, Urinary ,, ... ... ... | 24 |
| Cancer ... ... ... ... ... ... ... ... ... ... | 58 |
| Accidents and Miscellaneous Cases ... ... ... | 21 |
| Total ... ... | 426 |

This table merely shows the number of deaths under each head, as nearly as possible according to the set of organs in which the disease was seated, excluding however all forms of cancer, which, from the special interest attached to the subject, I have made into a separate class by itself; and very many *post mortem* examinations were made, especially in the earlier half of the time included in these returns.

TABLE No. III.

| General Diseases. | |
|---|---|
| Scarlatina ... ... ... ... ... ... ... ... | 9 |
| Typhoid ... ... ... ... ... ... ... ... ... | 8 |
| Smallpox .. ... ... ... ... ... ... ... ... | 1 |
| Typhus ... .. ... ... ... ... . ... ... | 1 |
| Cholera ... ... ... ... ... ... ... ... ... | 1 |
| Puerperal Fever ... ... ... ... ... ... ... | 2 |
| Gout ... ... ... ... ... ... ... ... ... ... | 1 |
| Melanosis ... ... ... ... ... ... ... ... | 1 |
| Tuberculosis ... ... ... ... ... ... ... ... | 1 |
| Marasmus or Atrophy ... ... ... ... ... ... | 5 |
| Old Age ... .. ... ... ... ... ... ... ... | 24 |
| Total ... ... | 54 |

In this list the proportion of deaths from zymotic diseases to the entire number of deaths is twice as great as that shown in the annual report of the medical officer of health for Bristol in the year 1887, which difference may be due to the fact that in his report there are, of course, very many other causes of death which do not occur in my practice; and my whole number is not large enough for a satisfactory comparison.

The case of cholera was that of the head master of the old Grammar School, in Unity Street, who was seized with his first symptom in the early morning of October the 14th, 1854, and died in the afternoon of the same day.

He was one of the victims of an outbreak of most virulent cholera in the district supplied by water from Jacob's Wells, which was afterwards found to be contaminated by sewage from Clifton; the special infection being most probably from a fatal case which had occurred a few days before in the immediate neighbourhood of Meridian Place.

I have never had a fatal case of measles.

The subject of melanosis was an unmarried lady, aged 34, who, two years before I saw her, when she was living in the North of England, had a small tumour removed by operation from the hip, near the great trochanter. She came to me complaining of swelling and pain in the lower part of the body; and upon examining her I found numerous black swellings, of various sizes from that of a shot to a broadbean, occupying the lower part of the abdomen and the external organs of generation. They increased very rapidly in number and size, extending down the thighs and over half the abdomen, and began to exude a fetid discharge, under which she soon sank. The appearance of the body after death was most remarkable.

Two of the cases of "marasmus or atrophy" were really cases of "debility from birth" in infants a few days old, in which there was not vitality enough to let the child live.

The cases returned as death from old age were those where there was no special disease capable of destroying life, but the patients sank away from senile feebleness; and yet there was in some a sign of some other ailment. Thus one is entered as "old age and herpes"; another as "old age and torpid bowels"; and sixty-seven of the entire number of the deaths were in persons above 80 years of age.

TABLE No. IV.

| Diseases of the Nervous System. | |
|---|---|
| Paralysis, Cerebral Softening, and Disease of the Base of the Brain ... ... ... ... ... | 19 |
| Epilepsy ... ... ... ... ... ... ... ... ... | 1 |
| Apoplexy ... ... ... ... ... ... ... ... ... | 25 |
| Tumour of the Brain ... ... ... ... ... ... | 5 |
| Meningitis ... ... ... ... ... ... ... ... | 5 |
| Paraplegia ... ... ... ... ... ... ... ... | 2 |
| Cerebritis (Acute) ... ... ... ... ... ... ... | 3 |
| Cerebritis (Chronic) ... ... ... ... ... ... | 8 |
| Dementia and Exhaustion ... ... ... ... ... | 2 |
| Hydrocephalus ... ... ... ... ... ... ... | 2 |
| Total ... ... | 72 |

The case of epilepsy was one of unusual character and interest. The patient was a poor basketmaker, in his 71st year, who had been epileptic since his 14th year, and quite blind and very deaf during that space of 57 years. He never had less than three fits in the day, often very many more; and when he felt the attack coming on, he would sit down on the floor with his back against the wall, and remain until he recovered himself. One day, instead of recovering, he remained insensible, and died in two days of apoplexy. At the *post mortem* examination I found the falx cerebri ossified in a thick mass, marked by the sulci and convolutions of the brain. In the upper part of the left hemisphere were two deposits of bone, like hard cancellous structure, as large as a nut, imbedded in the substance of the brain; another larger one, more deeply seated; and a fourth, which seemed to grow by a pedicle attached to the petrous bone. The ventricles were full of blood and clots, the immediate cause of death. This man probably had sixty thousand fits.

Two of the instances of tumour of the brain were in young children, where the disease seems to have followed an injury. The morbid growths (one as large as a filbert, the other the size of a walnut) were imbedded in the cerebral substance near the base, and they were formerly called tubercle of the brain ; now the word Glioma is used.

TABLE No. V.

| DISEASES OF THE CIRCULATING SYSTEM. | |
|---|---|
| Diseased Heart and Kidneys ... ... ... ... | 9 |
| Diseased Heart ... ... ... ... ... ... ... | 10 |
| Ditto with Dropsy... ... ... ... ... | 5 |
| Ditto with Syncope ... ... ... ... | 2 |
| Ditto with Anæmia ... ... ... ... | 1 |
| Embolism ... ... ... ... ... ... ... ... | 2 |
| Ruptured Aorta ... ... ... ... ... ... ... | 2 |
| Fatty Heart ... ... ... ... ... ... ... ... | 1 |
| Total ... ... | 32 |

Under the general head of "Diseases of the Heart," a diagnosis sufficiently precise for the registrar, are comprised cases of pericarditis with effusion, angina pectoris, pneumonia and pericarditis in the same subject, and a case of phlegmon and sphacelus of the leg from feeble heart-action ; and one case of sudden death in a lady, who had suffered from weak heart, was marked "palpitation of the heart," from what had been a prominent symptom before.

Respecting the nine cases of disease of the heart and kidneys, there is nothing recorded to determine what organ was first affected : that there are frequently heart-symptoms before there is any reason to suspect the kidney is quite clear ; and as the disease progresses the albumen suddenly makes its appearance.

In both the cases of rupture of the aorta, the blood escaped into the pericardium through a straight rent. Both were men, as is nearly always the case, and one was 58 and the other 70 years of age.

TABLE No. VI.

| Diseases of the Respiratory System. | |
|---|---|
| Tubercular Phthisis ... ... ... ... ... ... | 33 |
| Pleuro-pneumonia and Hydrothorax ... ... ... | 5 |
| Pneumonia ... ... ... ... ... ... ... ... | 21 |
| Bronchitis ... ... ... ... ... ... ... ... | 10 |
| Bronchitis Senilis ... ... ... ... ... ... ... | 27 |
| Emphysema ... ... ... ... ... ... ... ... | 2 |
| Croup ... ... ... ... ... ... ... ... ... | 1 |
| Asthma and Bronchitis ... ... ... ... ... ... | 4 |
| Diphtheria (Laryngeal) ... ... ... ... ... ... | 1 |
| Hæmoptysis ... ... ... ... ... ... ... ... | 3 |
| Contracted Trachea ... ... ... ... ... ... | 1 |
| Total ... ... | 108 |

These diseases are, in our climate, by far the most numerous and fatal of all. My proportion to the whole number is about one in four deaths; and in the registered returns for Bristol last year the proportion was rather greater, viz., one in three and a half; and in the numerous class, phthisis, the number was *thirty-three*, or one in every thirteen deaths—which is, within a very small fraction, the same rate as that which is returned by our medical officer of health for the whole district. A large number came under the head, "Senile Bronchitis;" and when I state that the average age of these twenty-seven persons was 78 years, it will be fair to consider that the "Anno Domini" had as much to do with the fatal result as the bronchitis.

Although many of the phthisical cases had the complication of occasional hæmoptysis in the usual progress of the malady, I have made a distinct class of the three cases which died in the act of bringing up blood. It is a terrible sight to stand by and witness a patient struggling to get his breath, while the rapid stream of florid blood is rushing from his mouth and nose, until he is speedily overpowered and suffocated, and to feel oneself perfectly powerless to help him.

The case of "contracted trachea" was not my patient originally. The subject of it had worn a tube in his windpipe for several years, and had suffered occasionally from difficult breathing during that time, apparently from narrowing of the trachea and extension downwards of the original disease of the larynx. I do not know if it was syphilitic, but it certainly was not cancer.

Pneumonia has been so common and so fatal a disease in my practice, that I always feel much anxiety about my cases, especially when there is evidence in the form of dulness and crepitus that both lungs are involved. Notwithstanding the preponderance of females in my general list, the number of deaths from pneumonia of males is nearly double that of females, because they are more exposed to cold and other influences likely to produce the complaint; and, besides seven children under five, they were all above 48 years of age—some of them men in the prime of life.

TABLE No. VII.

| Diseases of the Digestive System. | |
|---|---|
| Peritonitis and Inflammation of the Bowels ... | 8 |
| Diarrhœa ... ... ... ... ... ... ... ... ... ... | 5 |
| Infantile Diarrhœa ... ... ... ... ... ... ... | 5 |
| Perforation of the Intestine ... ... ... ... ... | 2 |
| Strumous Ulceration of the Bowels ... ... ... | 1 |
| Dyspepsia ... ... ... ... ... ... ... ... ... | 5 |
| Perforating Ulcer of the Stomach ... ... ... | 1 |
| Intestinal Obstruction ... ... ... ... ... ... | 2 |
| Intussusception ... ... ... ... ... ... ... | 2 |
| Ileus ... ... ... ... ... ... ... ... ... ... ... | 2 |
| Obstruction from Tumour ... ... ... ... ... | 2 |
| Thickened Pylorus... ... ... ... ... ... ... | 1 |
| Sphacelus of the Rectum ... ... ... ... ... | 1 |
| Imperforate Anus ... ... ... ... ... ... ... | 1 |
| Tabes Mesenterica ... ... ... ... ... ... ... | 4 |
| Chronic Hepatitis and Cirrhosis ... ... ... | 6 |
| Diseased Liver, with Jaundice and Dropsy ... | 7 |
| Abscess and Tumour of the Liver ... ... ... | 2 |
| Total ... ... | 57 |

Of these cases of peritonitis, one was caused by a tumour of the abdomen, probably cancerous ; and another, verified by *post mortem* examination, was a sudden attack in a gentleman aged about 50, coming on without any evident cause, unless it were cold from driving home at night in a damp cab. Another was a well-marked case of tubercular peritonitis.

In each of the cases of perforation of the intestine, the attack was very speedily fatal. One was male, the other female, both some way past the middle period of life, and in both the cause was excessive accumulation of hard fæcal matter in the bowels. In the case of the male there was a *post mortem* examination, and the whole large intestine was found blocked with heavy masses of hard and coherent fæces, which the peristaltic action of the bowels, aided by purgatives, was altogether unable to move; under such circumstances, the remedies only increased the perils of the patient.

The case marked "thickened pylorus" was in a young lady, generally active and bright, taken with persistent sickness; and at the *post mortem* examination, the only morbid condition to be found was a hard and thick state of the pylorus, which did not appear to be of a scirrhous nature.

One of the instances of intussusception was of a very remarkable and interesting kind. A healthy little boy, six years of age, was seized with violent abdominal pains, but was relieved, after a few hours, by a gentle purgative, which acted freely. Then the pain returned, and he was evidently suffering from ileus, or intussusception. He had a long illness of nearly six months, during which time he appeared once or twice to be moribund. He passed at times, *per anum*, sloughing pieces of intestine, and also passed gas and fæcal matter through the urethra. He was kept alive at first by port wine and beef tea, and the communication between the bladder and bowel ultimately healed, and his bowels were freely and tolerably regularly relieved; but the evacuation was slate-coloured and greasy, with undigested fat and no trace of bile. He took half a bottle of port wine, and plenty of nourishing food; but he gradually wasted away until every particle of flesh and fat was removed from his body, and he died practically of starvation, although able to feed well. At the *post mortem* examination the parts were found much matted together; but it was clearly made out that communication had taken place between the duodenum and descending colon by means of a double circular loop of bowel, and that the greater part of his alimentary canal had been unused for the whole time of his illness, thus explaining his want of nutrition; and the healed communication with the bladder had taken the form of a narrow and solid cord.

One case marked "Intestinal Obstruction" was that of one of our local Artillery Volunteers, who had severe attacks of obstruction occasionally after riding upon a rough and shaking gun-carriage, and at last the condition proved fatal. At the *post mortem* examination the only peculiarities were, that a large quantity of the small intestine had slipped down into the pelvis, and there was some little difficulty in withdrawing it, although there was no actual adhesion; and a pin which he had accidentally swallowed some years ago was found lying loose in the peritoneal cavity in the pelvis.

Some of these cases, had they occurred now, would have been considered suitable for operation, and it is possible that some might have been relieved.

The child with imperforate anus lived for 101 days, I believe, the longest time recorded that a child has lived without any evacuation. The usual vomiting ceased after a short time, and it took a little milk daily. I made first of all an unsuccessful attempt to reach the bowel, through the mark where the sphincter seemed to exist; and then the mother refused to have anything more done. Colotomy would, doubtless, have saved the child's life for a time; but, probably, the mother did the best thing when she objected to further surgical treatment. The rectum, from the sphincter to the bottom of the sigmoid flexure, was a mere solid fibrous band.

The case marked "Sphacelus of the Rectum" was a frightful one of neglected prolapsus ani, which was strangulated and began to slough; and then a rapid sphacelus attacked the sphincter and flesh of the nates, so that the still spreading sloughs hung down from the body of the patient, who suffered intolerable agony, unrelieved by any measures we could adopt.

TABLE No. VIII.

| Diseases of the Urinary System. | |
|---|---|
| Albuminuria ... ... ... ... ... ... ... ... ... | 5 |
| Ischuria renalis and Uræmia ... ... ... ... ... | 8 |
| Diabetes ... ... ... ... ... ... ... ... ... ... | 5 |
| Fungus Hæmatodes of the Bladder ... ... ... | 1 |
| Hæmaturia ... ... ... ... ... ... ... ... ... | 1 |
| Cystitis ... ... ... ... ... ... ... ... ... ... | 1 |
| Calculus Vesicæ (male) ... ... ... ... ... ... ... | 1 |
| ,, ,, (female ... ... ... ... ... ... | 2 |
| Total ... ... | 24 |

These albuminuria cases were for the most part closely allied to those in Table No. V., classed under the head "Diseased Heart and Kidneys," placed there because the heart-disease was the most marked condition; whilst in these others the state of the kidneys was more obviously the cause of death.

Of the deaths from diabetes, one was in a girl aged 18, in whom the disease ran through a most rapid course, and she wasted away and died after a very short illness, being conscious to the last breath. Another, a middle-aged lady, became suddenly comatose one day, and never recovered her senses; while a third died of a slowly progressing paralysis, affecting the use of the limbs and the power of speech.

The patient with fungus hæmatodes of the bladder had very much the same symptoms as those produced by stone in the bladder. He went up to London and saw the surgeons most celebrated for the treatment of such complaints, and was sounded by myself and others; but no one knew exactly what was the matter with him until I made a *post mortem* examination, and then I found a rough, ulcerated, bleeding fungoid surface, extending over exactly half the bladder, leaving the other half comparatively free from disease.

The male subject of calculus vesicæ was 73 years of age, who became my patient shortly before his death, his former surgeon having decided to give up the case. He was in a weak and almost sinking state, and there was no period after he was under my care when he was well enough to bear an operation. At the *post mortem* examination I removed a handful of stones, varying from the size of a pea to that of a filbert.

One of the female stone cases was 75 years old, and suffered much from bronchitis; and the other, aged 77, had diseased kidneys, and the urine was so full of phosphates that it was deposited in crusts about the external organs, and had to be removed occasionally; and the operation of clearing it all away was very painful.

The case of hæmaturia was stone in the kidney.

TABLE No. IX.

| CANCER. | |
|---|---|
| Cancer of the Lip ... ... ... ... ... ... ... | 1 |
| ,, Mouth ... ... ... ... ... ... | 1 |
| ,, Tongue ... ... ... ... ... ... | 2 |
| ,, Œsophagus ... ... ... ... ... | 3 |
| ,, Stomach ... ... ... ... ... ... | 1 |
| ,, Rectum ... ... ... ... ... ... | 4 |
| ,, Breast ... ... ... ... ... ... | 19 |
| ,, Uterus ... ... ... ... ... ... | 3 |
| ,, Vulva ... ... ... ... ... ... | 2 |
| ,, Eye ... ... ... ... ... ... ... | 1 |
| ,, Neck ... ... ... ... ... ... | 2 |
| ,, Brain ... ... ... ... ... ... | 1 |
| ,, Arm ... ... ... ... ... ... ... | 1 |
| Intestinal Cancerous Tumour ... ... ... ... | 7 |
| Unrecorded ... ... ... ... ... ... ... ... | 10 |
| Total ... ... | 58 |

In the registrar's returns for the city of Bristol for 1887, the deaths from cancer were one in every thirty-eight of the whole number of deaths: whilst in my own list they were as many as one in eight, or more than four times as frequent—so that I had more than my fair share of these most unsatisfactory cases. The proportion of the men to the women who died from this terrible malady under my care was about one man to three women, or one in four.

There is nothing very worthy of note respecting the ages at which death occurred: two were under ten years of age, none between ten and thirty-four, and then the number gradually increased with each decennial period; the number above seventy being greater than that of any other period of ten years.

My fatal case after operation for cancer of the lip, a proceeding generally considered to be entirely devoid of danger, was that of a gentleman, a dignitary of the Church, aged 72, on whom I operated in the usual way. The wound healed perfectly well, but a superficial sore line remained on the red part of the lip inside, and this was granulating and healing properly. He walked into Bristol one cold morning from the country-house where he was staying, and when he came back he had a rigor, followed by pyæmia and in a very few days by death.

Two out of three deaths from cancer of the œsophagus were in men; and I believe that the disease is more common in them than in women. It is very painful to treat these patients and to watch them day by day as they die of starvation. The female lived for a long time without any nourishment whatever, and became almost a skeleton in appearance. One of my male patients used to assuage the pangs of hunger, and of the complaint itself, by repeated doses of morphia, to which he had become so habituated that he took large quantities with impunity, but without much beneficial effect.

Patients with cancer of the rectum frequently live a long time after the disease is developed and recognised; and, as is the case with cancer of the other end of the alimentary tube, their sufferings are very great. In diseases of the rectum, whether cancerous or not, I have always noticed that there is a peculiar dread and anxiety, and an exaggerated idea of the amount of disease present, far more than is found in a corresponding amount of disease in any other organ.

With the exception of operations on the eyes, I have been called upon to perform operations on the female breast for cancer or other tumours more often than any other, in private practice. I do not remember to have had a fatal case among the many operations of this kind that I had at the Bristol Infirmary during the twenty years to which my term of office as Surgeon was, by the rules then in force, restricted; but among the cases brought together in this table under the head of "Cancer of the Breast," no less than three such deaths occurred, the particulars of which I wish briefly to record.

The first was an undoubted case of pyæmia. The patient was nearly well, and the wound healed all but a small, healthy-looking, granulating surface, when she had a rigor, the precursor of fever, and deposits of pus in the joints and tissues, and speedy death, from what used to be called "absorption of pus." At the present time a free use of disinfectants might possibly have prevented such a sad end to a very promising case.

The second death occurred in the person of a lady living a short distance out of Bristol, and took place about the time of the first introduction of carbolic acid as a disinfectant. I took over and left in the house a four-ounce bottle of pure carbolic acid, that I might put a few drops in the water when the wound was dressed and the part bathed. The case went on fairly well, but there was a good deal of suppuration; and one night, as the patient complained of the smell of the dressings, the nurse saturated a piece of cotton wool with my strong acid, and placed it between her arm and her side, just below the site of the wound. When I came in the morning there was a long slough of the skin on the side below and parallel with the wound, and another on the forearm, and the patient was very sick and ill, poisoned, I imagine, by the carbolic acid. The original wound opened and ceased to discharge, and looked dry, so that I could see the exposed pectoral muscle at the bottom of it; and the wound caused by the acid sloughed, and the patient died in a few days. I do not know whether it was pyæmia or carbolic acid poisoning; but I never saw a wound in the state this one was in, quite dry and as open as it was immediately after the removal of the tumour.

The third death after the removal of a cancerous tumour of the breast was of a still more unusual character, and I have never heard of a similar case. The wound healed chiefly by first intention, and there was promise of a speedy convalescence, when one day the patient told me that she had taken cold and had pain in her face. The next day the muscles of the face were stiff and hard, and there was unmistakeable "risus sardonicus," and pain in the abdominal muscles. These symptoms developed rapidly into acute tetanus of a most violent kind, with opisthotonos and all the other signs, as severe in this delicate lady as ever I saw in the case of a strong man at the Infirmary, and with the usual end of such acute cases, for death soon relieved her from her sufferings. I discovered afterwards that this patient had various small melanotic tumours about her body.

I should like to know whether the present free use of disinfectants has lessened the number of tetanus cases.

Of the other deaths from cancer of the breast, some were instances of the recurrence of the disease after operation, two from repeated hæmorrhages, and in some the patients had concealed their malady until any surgical interference became impossible: and if I could give the number and the fate of all the cases of the disease about which I have been consulted, I fear that the list of fatalities would be very long.

One family appears several times in this sad catalogue. A daughter from whom I had removed a cancerous breast died, aged 51, after a tedious and very painful illness, from a recurrence of the disease. The house was small, and the fetor from the large sore was most offensive and pervaded the place. After a considerable time the mother had symptoms of disease of the uterus, and she died from repeated hæmorrhages from a large and ulcerated cancerous uterus, at the age of 75. Then the father died, aged 80, from a large internal tumour; and the youngest daughter followed, at the age of 37, with cancer in the breast and stomach. There are now three surviving sisters of this cancer-stricken family; and although, perhaps, only one of the innumerable coincidences we meet with, I think it worth mentioning that neither of the three was living in the house when the first case occurred, and they were not exposed to the effluvium I have mentioned; and also that they are, I believe, free from the disease, the eldest, a teetotaller, being 70 years of age; and that they have all three been persons who have indulged very freely in alcoholic drinks.

Cancer of the vulva is one of the most painful and distressing forms of the disease; and in one of these cases it recurred after repeated operations, and the patient died after suffering great tortures.

The two cases of cancer in the neck were like one another, and appeared to begin by enlargement and induration of the cervical glands, gradually increasing until the tumour occupied the space between the jaw and the clavicle, and the skin ulcerated and sloughed, causing a large unhealthy wound which discharged blood and serum and pus having the peculiar sickening odour of cancer, lasting for many weeks, until, no vital part being touched by the disease, the strength of the patients was simply worn out.

The case marked "Cancer of the Brain" was a patient from whom I had removed a cancerous breast, and the disease re-appeared in the brain. I had a similar case, which does not appear in these notes, at the Infirmary, when the patient made a good recovery from the operation and went out, and became stout and plethoric and apparently well; but she died suddenly from apoplexy, the hæmorrhage coming from a cancerous deposit in the brain.

TABLE No. X.

| Accidents and Miscellaneous Cases. | |
|---|---|
| Overlaid .. ... ... ... ... ... ... ... ... | 1 |
| Sphacelus of the Leg... ... ... ... ... ... | 2 |
| ,, Hand and Arm ... ... ... | 1 |
| Fractured Skull ... ... ... ... ... ... ... | 1 |
| ,, Thigh ... ... ... ... ... ... ... | 2 |
| ,, Neck of Femur ... ... ... ... ... | 1 |
| Caries of the Spine ... ... ... ... ... ... | 2 |
| Curvature ,, ... ... ... ... ... ... | 1 |
| Diseased Hip Joint ... ... ... ... ... ... | 1 |
| Ovarian Tumour ... ... ... ... ... ... ... | 3 |
| Cranial Tumour ... ... ... ... ... ... ... | 1 |
| Anthrax (Pyæmia)... ... ... ... ... ... ... | 1 |
| Unrecorded ... ... ... ... ... ... ... ... | 4 |
| Total ... ... | 21 |

These cases require but little comment.

The two instances of sphacelus of the leg were old men, and both had been seafaring men all their lives until they retired from active work; one of them a man of note, of a much-respected and well-known name. The cause of the sphacelus was weakness of the circulation in the legs, I think from atheroma of the arteries; and one of them went through an acute and rapid course to the end, while the other was chronic. These cases of mortification of the leg, as they are usually called, are by no means uncommon.

The fractured skull was the case of a schoolboy aged 14, who fell over the banisters and pitched on his vertex on a stone floor. His skull was split longitudinally, so that a definite depression could be felt along the top of his head, the line of fracture. He died in a few hours.

In my practice, fracture of the neck of the thigh-bone has not proved by any means a fatal injury. The patient whose death is here recorded was 94 years of age.

Both the cases of spinal caries died from exhaustion from the discharge of psoas abscess.

Anthrax is credited with one of the deaths in this table. It was really a death from chronic pyæmia. The patient, a clergyman aged 60, was sent to be under my care from a distance, suffering from the effects of a carbuncle which had been incised* and was nearly healed; but he had abscesses in various parts of the body, the result of undoubted blood-poisoning from the original disease. The case was chronic, but hopeless from the first.

To conclude: this task of writing a record or *resumé* of between four and five hundred fatal cases, without a single success among them, although you are conscious that it represents very many thousands of successful ones treated at the same time, is a depressing work, and in some portions of it, such as that dealing with those terrible cancer cases, it is a pain to remember them all; and also it is impossible to drive away the question which keeps intruding itself, Has the best possible been done for all these cases?

* I have elsewhere recorded my opinion about the use of the knife in carbuncle, and wish here to avoid all discussion as to treatment.

My record begins more than forty years ago, and it is said, I believe truly, that never has our art improved so rapidly in a corresponding space of time as it has in the last fifty years. Therefore, judging from this point of view, and seen by the light of our present knowledge, some of these lives now might be saved, or at any rate prolonged. Take, for example, the treatment of cases of cancer of the œsophagus by gastrotomy; the removal of ovarian tumours; the abdominal section for intestinal obstruction; and nephrectomy; and also the power, with the aid of anæsthetics and antiseptics, of performing other operations previously impossible: and again, in the domain of pure medicine, valuable new remedies have been discovered, which, with new and better treatment founded on more accurate pathology, have doubtless made our efforts more successful in saving or prolonging life, or relieving some of the vast amount of suffering we are called upon to witness.

I must draw the line here, and not venture any further into this enticing subject.

